# Supplementary material for: Setting benchmarks for modelling gas–surface interactions using coherent control of rotational orientation states
Source: Nat Commun. 2020 Jun 19;11:3110. doi: 10.1038/s41467-020-16930-1 (PMC7305202; doi:10.1038/s41467-020-16930-1)
Supplement: Supplementary file 1 — Supplementary Information [file 41467_2020_16930_MOESM1_ESM.pdf]

Supplementary Information

Setting benchmarks for modelling gas-surface interactions using coherent control of rotational orientation states.

Alkoby et al

Supplementary Note 1

Signal Calculation Methods

The starting point of the calculation is a molecular beam with equal population in each of the nine m_I, m_J states. The beam enters a magnetic hexapole[1], where the molecules experience different quantised forces determined by their magnetic moment[2,3]. Semi-classical trajectory calculations are used to find the probability that each m_I, m_J state is transmitted through the first hexapole ($P_{\mathrm{hex1}}(n)$, where n denotes the initial m_I, m_J state). At the end of the hexapole, there is a hexapole to dipole element which adiabatically rotates the magnetisation from the plane of the hexapole to the direction of the initial quantisation axis, denoted Z in Supplementary Figure 1. As the superposition states decohere due to the strong magnetic field gradients[2] this produces nine different populations each corresponding to a pure Ψ_{0n} where the relative populations are given by $P_{\mathrm{hex1}}(n)$.

The molecules are propagated through the rest of the machine using a mixed classical-quantum approach, in which the center-of-mass motion of the molecule is treated classically and the internal (nuclear-spin and rotational) degrees of freedom are treated quantum mechanically. The Hamiltonian governing the evolution is

$$H(B) = \frac{\hbar^2 k^2}{2m} + H_{\mathrm{R}}(B) \tag{1}$$

where the first term is the molecule's center-of-mass kinetic energy, and $H_{\mathrm{R}}(B)$ is the Ramsey Hamiltonian given by[4]

$$\frac{H_{\mathrm{R}}(B)}{h} = -a\frac{\mathbf{I}\cdot\mathbf{B}}{B} - b\frac{\mathbf{J}\cdot\mathbf{B}}{B} - c\,\mathbf{I}\cdot\mathbf{J} + d\left(3\,(\mathbf{I}\cdot\mathbf{J})^2 + \frac{3}{2}\,\mathbf{I}\cdot\mathbf{J} - \mathbf{I}^2\mathbf{J}^2\right) \tag{2}$$

The parameters a, b, c and d account for the relative weights of the different terms[4]. The first two terms are magnetic field dependent ($a \propto B$ and $b \propto B$) and account for the interaction of the nuclear spin (**I**) and rotational angular momentum (**J**) with the applied magnetic field (**B**). The third term accounts for the magnetic spin-rotation interaction, and for H_2 the fourth term accounts for the spin-spin interaction between the two nuclei[4]. These final two terms are

independent of the magnetic field, meaning it is also necessary to propagate the wave function through regions of space where there are no applied magnetic fields.

Time evolution calculations of the internal magnetic quantum states which adiabatically follow the molecular centre-of-mass trajectories are used in the first and second arms of the machine. The coherent propagation of Ψ_{0n} through the first arm is started at a point where the dipole field in the Z direction is still sufficiently large that the nine initial m_I, m_J states are pure states[2]. Each m_I, m_J state is then propagated individually through a three dimensional magnetic field profile that characterises the fields the molecules experience when travelling down the first arm using the Hamiltonian given in Supplementary Equations 1 and 2. In an ideal experiment, these profiles would describe the rest of the dipole field which is directed along Z, the zero field region between the end of the dipole and the start of the solenoid, the solenoid field which is directed along –X with magnitude B1 and the zero field region between the end of the solenoid and the surface. However, to obtain a realistic simulation of the experiment, the profiles we used for the calculation also include small (< 0.1 gauss metre) residual magnetic fields which were carefully characterised throughout our setup. By propagation through these fields, the initial pure state $|n\rangle$ becomes a coherent superposition of the nine m_I, m_J states as the population of the different states undergo Rabi oscillations. Propagation of the states through the first arm of the machine can be expressed using a matrix $U(B1)$, which is calculated using the methods described in a previous publication[5]. Therefore, the wave function of the molecules before they hit the surface using Z as the quantisation axis can be written as $\Psi_{1n}^{Z} = U(B1)\sqrt{P_{\mathrm{hex1}}(n)}|n\rangle$.

The quantisation axes of scattering matrices (S) are typically taken to be the surface normal[6]. This makes it necessary to rotate Ψ_{1n}^{Z} so that the quantisation axis is Z_N (see Supplementary Figure 1) which gives $\Psi_{1n}^{Z_N} = R(\theta_1)\Psi_{1n}^{Z} = R(\theta_1)U(B1)\sqrt{P_{\mathrm{hex1}}(n)}|n\rangle$, where θ_1 is the angle between Z and Z_N. The wave function is then scattered from the surface, with the change characterised by the scattering matrix (S-matrix). The wave function after scattering can be written as $\Psi_{2n}^{Z_N} = SR(\theta_1)U(B1)\sqrt{P_{\mathrm{hex1}}(n)}|n\rangle$, using the surface normal as the quantisation axis. As the propagation down the second arm of the machine is done with respect to the Z' axis this has to be rotated to give $\Psi_{2n}^{Z'} = R(\theta_2)SR(\theta_1)U(B1)\sqrt{P_{\mathrm{hex1}}(n)}|n\rangle$, where θ_2 is the angle between Z_N and Z'.

The wave function is then propagated through the three dimensional magnetic field profile that includes the zero field region between the surface and the start of the solenoid in the second arm, the magnetic field of the solenoid with magnitude B2 that is directed along the –X' direction, the zero field region between the end of the solenoid and the dipole which defines the –Z' quantisation axis, and as far into the dipole as is necessary for the field to be large enough to no longer mix the states. Analogously to the first arm, this propagation is described by the evolution matrix $U(B2)$. The wave function at the start of the second hexapole is given by $\Psi_{3n}^{Z'} = U(B2)\ R(\theta_2)SR(\theta_1)U(B1)\sqrt{P_{\text{hex1}}(n)}|n\rangle$.

The probabilities that the molecules in a final state f are transmitted through the second hexapole ($P_{\text{hex2}}(f)$) are again calculated using semi-classical trajectories. The signal from the detector is given by $\sum_f \sum_n \langle \Psi_{fn}^{Z'} | \Psi_{fn}^{Z'} \rangle$, where

$$\left|\Psi_{fn}^{Z'}\right\rangle = \sqrt{P_{\text{hex2}}(f)}\ U(B2)\ R(\theta_2)\ S\ R(\theta_1)\ U(B1)\ \sqrt{P_{\text{hex1}}(n)}|n\rangle \tag{3}$$

Strictly the discussion presented above gives the signal for a molecular beam where the molecules are travelling at a single velocity for a single B1 and B2 value. The experiments presented in the manuscript were performed for two values of B2 and 301 B1 values, and the calculation of both $U(B1)$ and $U(B2)$ is repeated for all the magnetic fields where the experimental signal is measured. In addition, the process is repeated for a range of velocities which is considered to represent the velocity width of the molecular beam that contributes to the signal, and the final calculated signal is taken as the weighted average of the signals for the different velocities.

The signal can also be calculated using a fully quantum mechanical approach which has been described elsewhere[7]. It has been confirmed that these two different approaches to calculating the signal produces the same result at the values of the magnetic fields that are used in the current work.

Supplementary Note 2

Data Analysis

The method outlined above also allows empirical scattering matrices to be determined by fitting the experimental data, as the only unknown in Supplementary Equation 3 is the S-matrix. This is given as

$$S = \begin{pmatrix} s_{11}e^{ik_{11}} & s_{10}e^{ik_{10}} & s_{1-1}e^{ik_{1-1}} \\ s_{01}e^{ik_{01}} & s_{00}e^{ik_{00}} & s_{0-1}e^{ik_{0-1}} \\ s_{-11}e^{ik_{-11}} & s_{-10}e^{ik_{-10}} & s_{-1-1}e^{ik_{-1-1}} \end{pmatrix} \quad (4)$$

where s_{fn} are the amplitudes for a transition from an initial m_J state n to a final m_J state f, and k_{fn} are the associated phases.

The number of amplitudes that are used in the S-matrix can be reduced by considering the symmetry of the scattering problem. The LiF surface has reflection symmetry along the [110] direction, which corresponds to the direction of the (1,0) and (-1,0) diffraction peaks considered here. This leads to the interaction potential also being symmetric. To be real, the collision has to be symmetric with respect to reflection in the scattering plane (along the [110] direction). As the reflection changes the projection of molecules that are in m_J = 1 to m_J = -1 (and vice versa), this places constraints on the S-matrix as given below.

$$S = \begin{pmatrix} s_{11}e^{ik_{11}} & s_{10}e^{ik_{10}} & s_{1-1}e^{ik_{1-1}} \\ s_{01}e^{ik_{01}} & s_{00}e^{ik_{00}} & s_{01}e^{ik_{0-1}} \\ s_{1-1}e^{ik_{-11}} & s_{10}e^{ik_{-10}} & s_{11}e^{ik_{-1-1}} \end{pmatrix} \quad (5)$$

This constrained version of the S-matrix was used in the fitting procedure. In this S-matrix, the sum of the square of the amplitudes in the first and third columns are the same, which means that the (relative) populations in m_J = 1 and m_J = -1 that scatter into the diffraction channel are the same. Likewise, the sum of the square of the amplitudes in the first and third rows are the same, meaning that the (relative) populations in m_J = 1 and m_J = -1 after scattering are the same.

To fit the data, the matrix $U(B1)$ is calculated for propagating the nine initial m_I, m_J states through the first arm of the machine for the values of B1 used experimentally, and for the range of velocities in the molecular beam that contribute to the signal. A $U(B2)$ matrix is also

calculated for the values of B2 that are used in the measurements and for the same velocity distribution. Combining these with the probabilities that the different states are transmitted through the two hexapoles and the rotations using Supplementary Equation 3 allows a signal to be calculated for a given S-matrix. As there is an unknown background component in the experimental signal, the measured intensities for two different values of B2 are fitted simultaneously assuming that the background is the same in both measurements. The difference between the experimental data and calculated signal is minimised using the downhill simplex method of Nelder and Mead[8] in combination with simulated annealing to ensure the algorithm finds the global minimum.

Supplementary Note 3

Convergence of the Optimal S-matrix

To ensure convergence of the empirically determined S-matrix, 150 signal calculations were run for each diffraction peak with randomly sampled initial values of all the amplitudes s_{fn}, and phases k_{fn}. The results of this are presented in Supplementary Figure 2 which shows the best fits for the (1,0) (panels a and b) and (-1,0) peaks (panels c and d) for the two values of B2 used in the simultaneous fits, Supplementary Figure 3 which shows the values of s_{fn} obtained from the 30 best fits for the (-1,0) diffraction peak, and Supplementary Figure 4, which shows the phases k_{fn} for the same peak. This demonstrates the convergence of the fits and that a single S-matrix is obtained by fitting the data. It should be noted that as we don't have a measure of the absolute flux that is scattered into a diffraction channel, the analysis gives relative values of the amplitudes of the S-matrix elements, and not the absolute values. The signal is also only sensitive to phase differences between the phases of the different S-matrix elements, so the analysis only determines the relative phases.

As a further illustration of the convergence of the fits, Supplementary Figure 5 presents the ratios of molecules with helicopter type rotation to those with cartwheel type rotation, quantified as $m_J = 1/m_J = 0$, as a function of the final error between the experimental data and the fit. This is shown for the (1,0) diffraction channel before (panel a) and after (panel b) the collision, and for the (-1,0) diffraction channel before (panel c) and after (panel d) the collision. For the best fits (those with the smallest error), the polarising and analysing properties of the two diffraction channels are consistently obtained from the fitting procedure.

Supplementary Note 4

Accuracy and Reproducibility of S-matrix Determination

While the results mentioned above show that the analysis converges on a single S-matrix, we can use a simulated signal to verify that the convergence actually coincides with the correct S-matrix. For this we generated simulated signals, from a known S-matrix, which included noise levels consistent with those in the experimental measurements. An optimal S-matrix was obtained from these simulated signals using exactly the same analysis procedure used for the experimental data. The simulated signal and the best fits to the data are presented in Supplementary Figure 6 for the 0 gauss metre simulated data (panel a) and the 558 gauss metre simulated data (panel b). The S-matrix amplitudes from the best fits are presented in Supplementary Figure 7 and the phases in Supplementary Figure 8, with the black dashed lines in each panel showing the value of the parameter that was used to simulate the data. Again it should be emphasised that these are relative S-matrix amplitudes and phases relative to s_{10} and k_{10}, and not absolute values. As can be seen, the fitting procedure converges on the values of the parameters that were used to generate the simulated signal. Taking the average value of the parameters obtained from the best 30 fits (lowest fitting error) gives values that are within 10% of the values that were used to simulate the signal. This demonstrates that the noise in the experimental data is small enough to not hinder our ability to extract an empirical S-matrix from the data.

There are also several systematic errors which can affect the accuracy of the empirical scattering matrix extracted using our analysis procedure, the first of which is the accuracy of the 3d magnetic field profiles that are measured for the first and second arm of the machine. These have been determined with a calibrated high precision gaussmeter (AlphaLab Vector Gauss Meter) that is accurate to ±0.02 gauss and 1% of the reading. In addition to fitting the data with the nominal magnetic field profile, we have also calculated the propagation of the wave functions through magnetic field profiles which include the maximum error these uncertainties introduce into the measurements. These fits change the S-matrix values by a few percent, but do not affect the conclusions of whether more helicopters or cartwheels scatter into the diffraction channel, or whether more helicopters or cartwheels are observed after scattering.

An additional uncertainty related to the magnetic field profiles is the conversion from the current that is passed through the solenoid to the magnetic field that it produces. This has been

determined by measuring the profile of the field in both solenoids at a known current. The profile was integrated, and a conversion calculated which allows the magnetic field to be calculated for the experimentally applied current for both solenoids. The sufficient accuracy of this calibration for the first arm is demonstrated by the fact that the measured signal and the calculated signal have the same oscillation frequencies. For the second arm, a 1% error in the current to magnetic field conversion changes the calculated signal by less than the errors in the experimental measurement. This means that any residual errors in these calibrations should not significantly affect the analysis of the data or the conclusions that are obtained.

While we use a beam flag to subtract the H_2 background in the particle detector, there is also an unknown constant background component in the beam contribution to the measured signal. This is due to the para-hydrogen content of the molecular beam and a certain fraction of hydrogen molecules which do not follow the adiabatic and non-adiabatic transitions and therefore cannot be coherently controlled by the B1 and B2 fields. In order to assess and separate this background signal from the part of the signal that characterises the state-to-state scattering, we fit two signals simultaneously for different values of B2, as we expect a similar background for the two measurements. This is justifiable, as any mechanism that produces the background should be independent of the value of B2, and the two measurements were done under the same experimental conditions (e.g., nozzle temperature, backing pressure, etc). It is not possible to extract a reliable S-matrix from only one measurement with an unknown background, as different S-matrix values can be found with different backgrounds which give equally good fits to the data.

Finally, another unavoidable source of error in our analysis procedure is related to the use of a single S-matrix for the different velocities in the molecular beam, rather than velocity dependent S-matrix elements. To minimise the effect of this necessary simplification, we chose to perform all the measurements on the diffraction peaks, rather than on the specular peak. While we lose signal intensity, the angular position of the diffraction peaks depends on the velocity of the molecules, and the very high angular resolution of our setup dramatically reduces the width of the velocity distribution contributing to the signal. In the measurements presented in the manuscript this width is assessed as 0.5% FWHM. The very good fit quality suggests that this simplification is reasonable and a single S-matrix approach can be used for a beam of scattered molecules with a very narrow energy distribution.

Parameter	(1,0) peak	(-1,0) peak	Parameter	(1,0) peak	(-1,0) peak
s_{11}/s_{1-1}	0.4	0.2	$k_{11} - k_{1-1}$	0.0	1.4
s_{10}/s_{1-1}	0.8	0.7	$k_{10} - k_{1-1}$	0.8	1.9
s_{1-1}/s_{1-1}	1.0	1.0	$k_{1-1} - k_{1-1}$	0.0	0.0
s_{01}/s_{1-1}	0.3	0.6	$k_{01} - k_{1-1}$	5.6	4.2
s_{00}/s_{1-1}	0.5	0.2	$k_{00} - k_{1-1}$	6.0	0.9
s_{0-1}/s_{1-1}	0.3	0.6	$k_{0-1} - k_{1-1}$	3.3	0.9
s_{-11}/s_{1-1}	1.0	1.0	$k_{-11} - k_{1-1}$	1.7	5.1
s_{-10}/s_{1-1}	0.8	0.7	$k_{-10} - k_{1-1}$	0.0	0.1
s_{-1-1}/s_{1-1}	0.4	0.2	$k_{-1-1} - k_{1-1}$	2.2	5.6

Supplementary Table 1. The relative values of the amplitudes (s_{fn}) and phases (k_{fn}) of the S-matrix elements from the best fits for H_2 scattering from LiF into the (1,0) and (-1,0) diffraction peaks. These values are averaged over the 30 fits which give the lowest fitting error. The estimated uncertainty in these values is 10%.

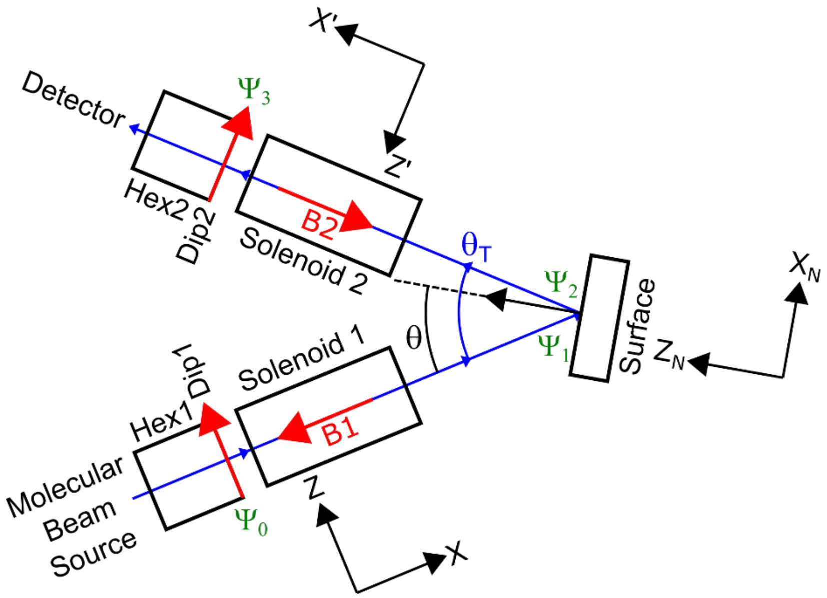

Supplementary Figure 1. Overview of the experimental apparatus. Schematic overview of the machine showing the frame of reference for the first and second arms, as well as at the surface (black arrows, in all cases the Y-axis is directed into the plane of the page), the directions of the magnetic fields (red arrows), the propagation direction (blue arrows), the angle between the two arms of the machine ($\theta_T = 45°$) and the incident angle with respect to the surface normal, θ. $\Psi_0 \longrightarrow \Psi_3$ denote wave functions at various points of the machine and are defined in the text.

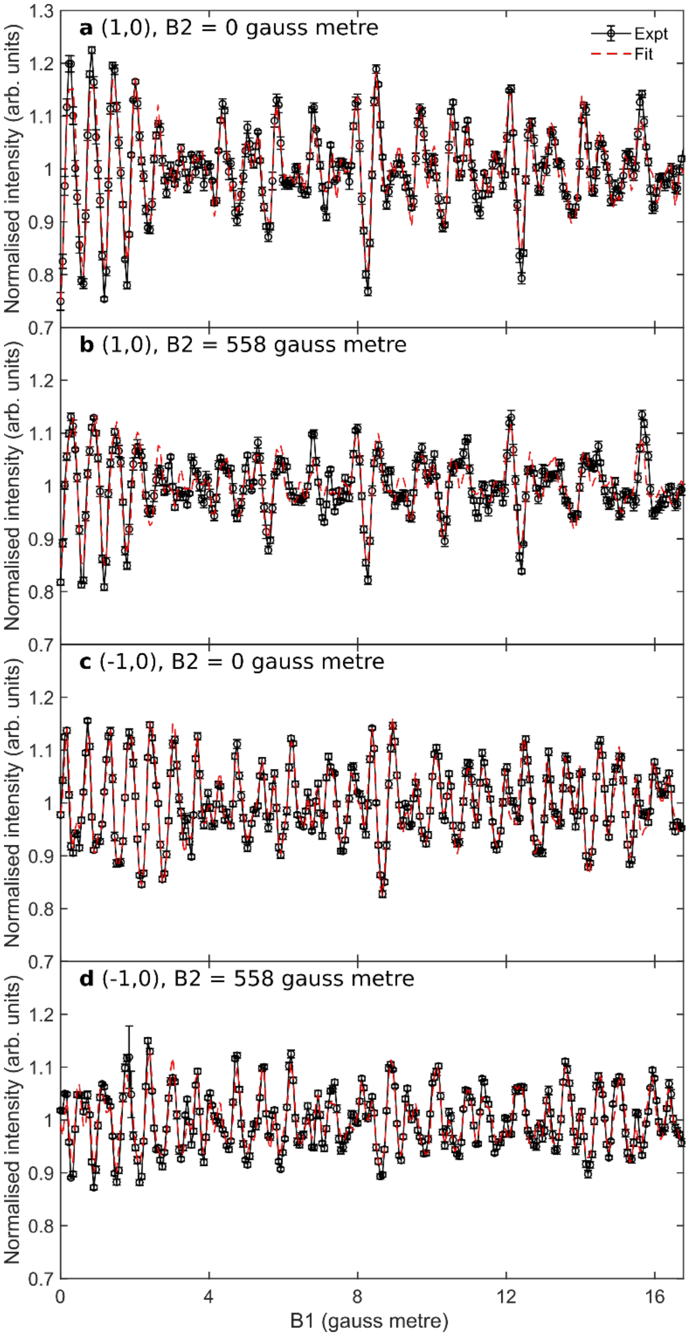

Supplementary Figure 2. Fitting experimental data with multiple B2 values. The experimental signal (black circles) normalised to the mean and the corresponding fits (red dashed lines) for the (1,0) peak measured at a value of B2 of 0 gauss metre (**a**) and 558 gauss metre (**b**), and the normalised experimental signal and fits for the (-1,0) peak for values of B2 of 0 gauss metre (**c**) and 558 gauss metre (**d**). The error bars represent standard errors from repeated B1 scans. The simultaneous fit of oscillation curves measured for two different B2 values allows us to identify the constant background contribution and extract a single S-matrix from the fit.

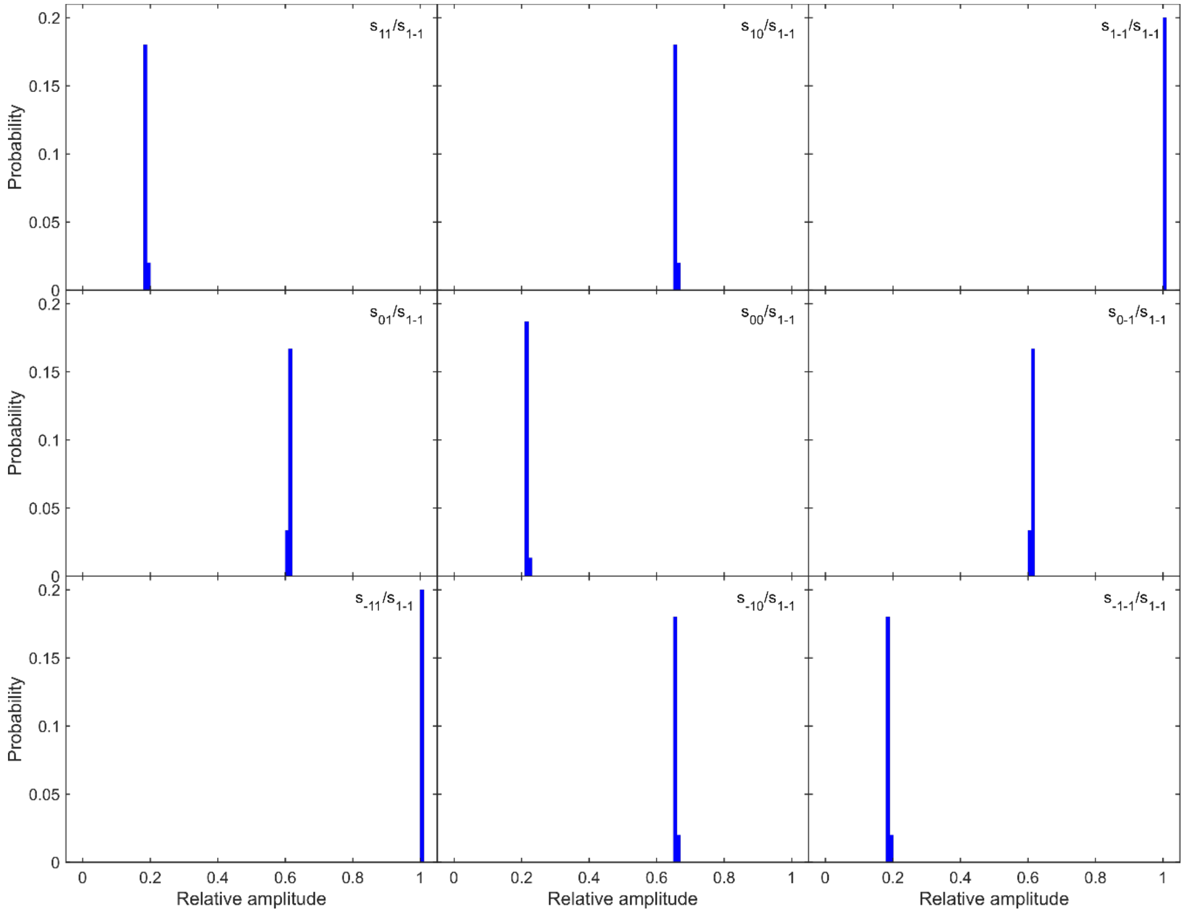

Supplementary Figure 3. Convergence of the empirical S-matrix amplitudes. The values of the S-matrix amplitudes (normalised to s_{1-1}) obtained from the best 30 of 150 fits of the (-1,0) diffraction peak data presented in Figure 3b. The distributions are narrow, demonstrating the convergence of the 15 parameter fits, and a relatively small uncertainty in the extracted value.

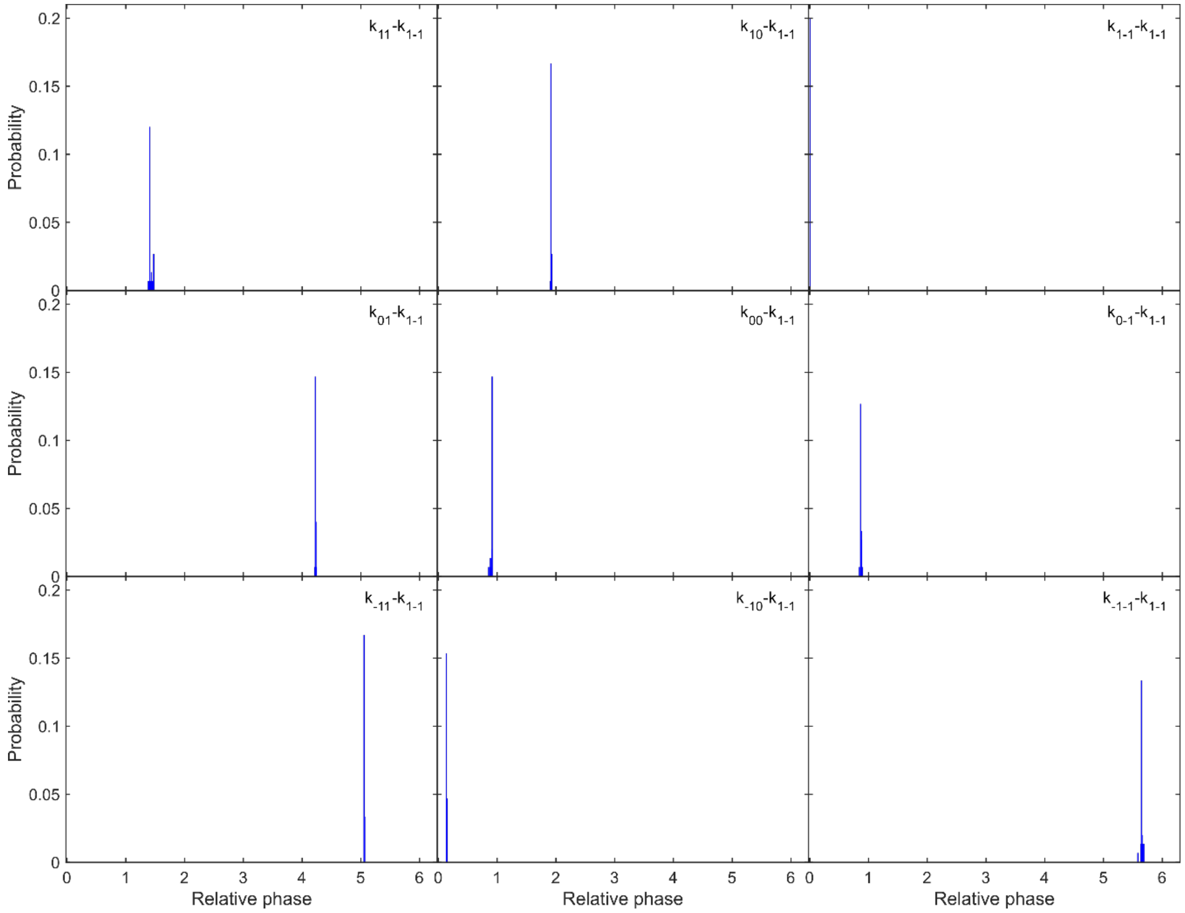

Supplementary Figure 4. Convergence of the empirical S-matrix phases. The values of the S-matrix phases (relative to k_{1-1}) obtained from the best 30 of 150 fits of the (-1,0) diffraction peak data presented in Figure 3b. The distributions are narrow, demonstrating the convergence of the 15 parameter fits, and a relatively small uncertainty in the extracted value.

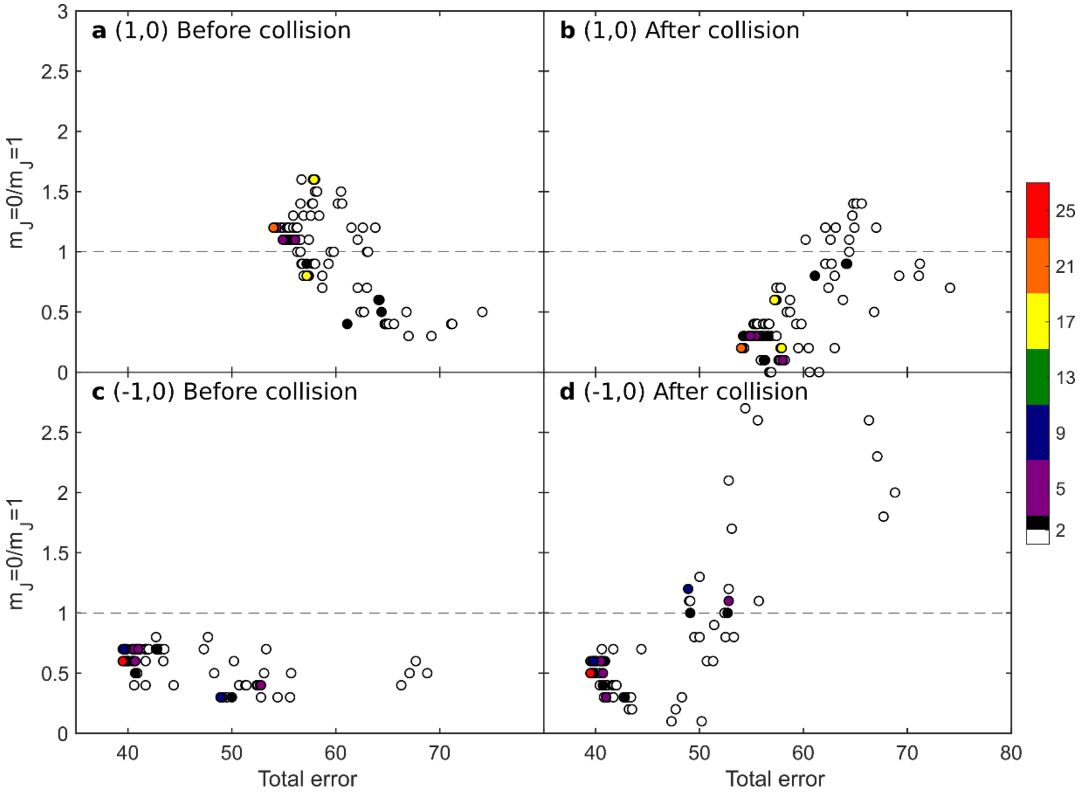

Supplementary Figure 5. Cartwheel to helicopter ratios before and after scattering. The ratio of cartwheels ($m_J = 0$) to helicopters ($m_J = 1$) determined from the empirical scattering matrices as a function of fitting error from 150 fits for the (1,0) diffraction channel before scattering (**a**), the (1,0) diffraction channel after scattering (**b**), the (-1,0) diffraction channel before scattering (**c**) and the (-1,0) diffraction channel after scattering (**d**) as a function of the total fitting error. The data have been histogram binned to the nearest 0.1 on both axes, and the colors represent the number of fits that fall into the bin, as given by the color bar. The dashed lines mark equal probabilities of $m_J = 1$ helicopter and $m_J = 0$ cartwheel states.

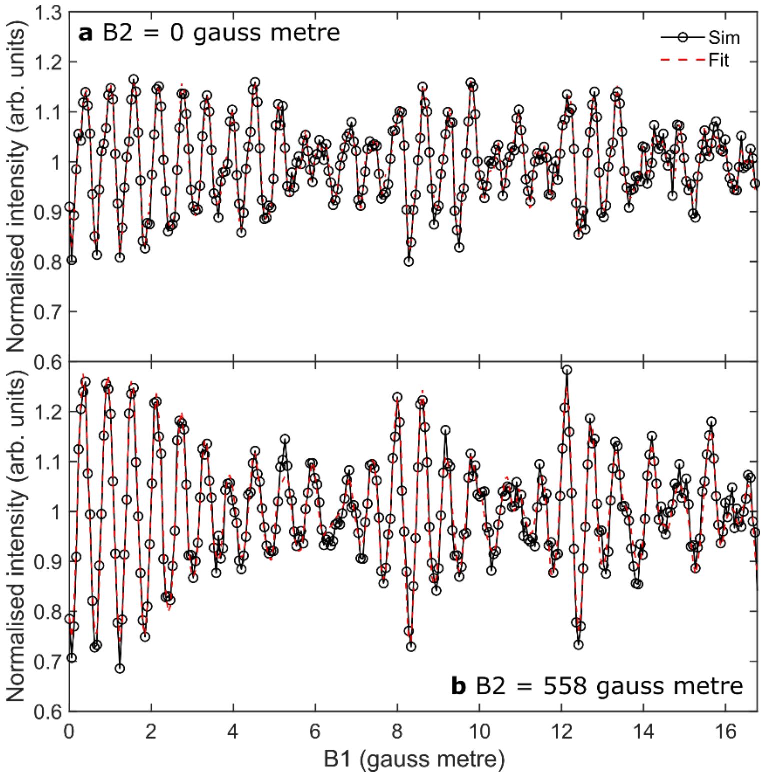

Supplementary Figure 6. Fits to simulated data. The best fits (red dashed line) to simulated signals (black line) for values of B2 = 0 gauss metre (**a**) and B2 = 558 gauss metre (**b**).

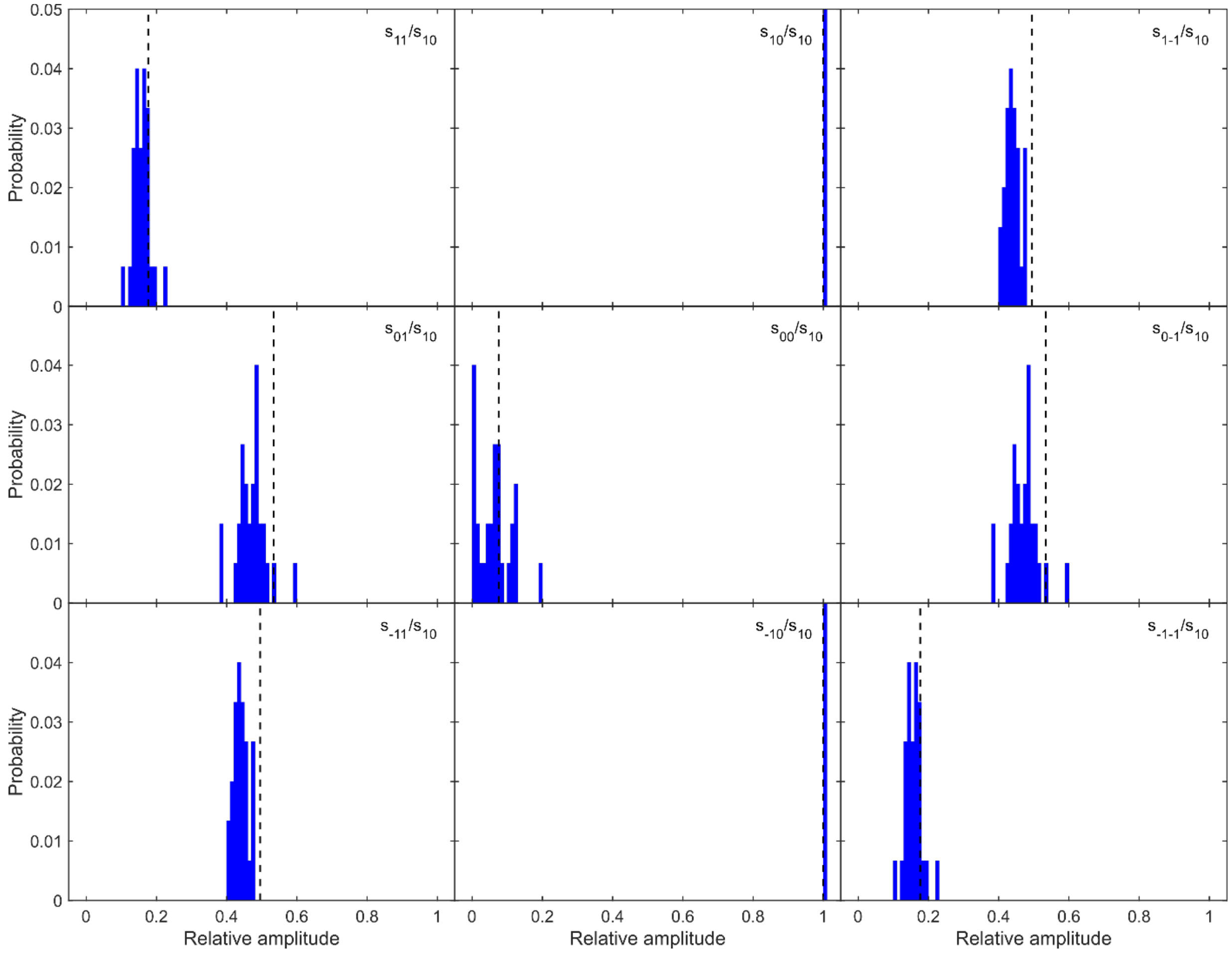

Supplementary Figure 7. Convergence of S-matrix amplitudes. The values of the S-matrix amplitudes (normalised to s_{10}) obtained from the best 30 of 150 fits of the simulated data presented in Figs. S6a and S6b, demonstrating the convergence of the 15 parameter fits. The black dashed line in each panel shows the value of the parameter that was used to produce the simulated data.

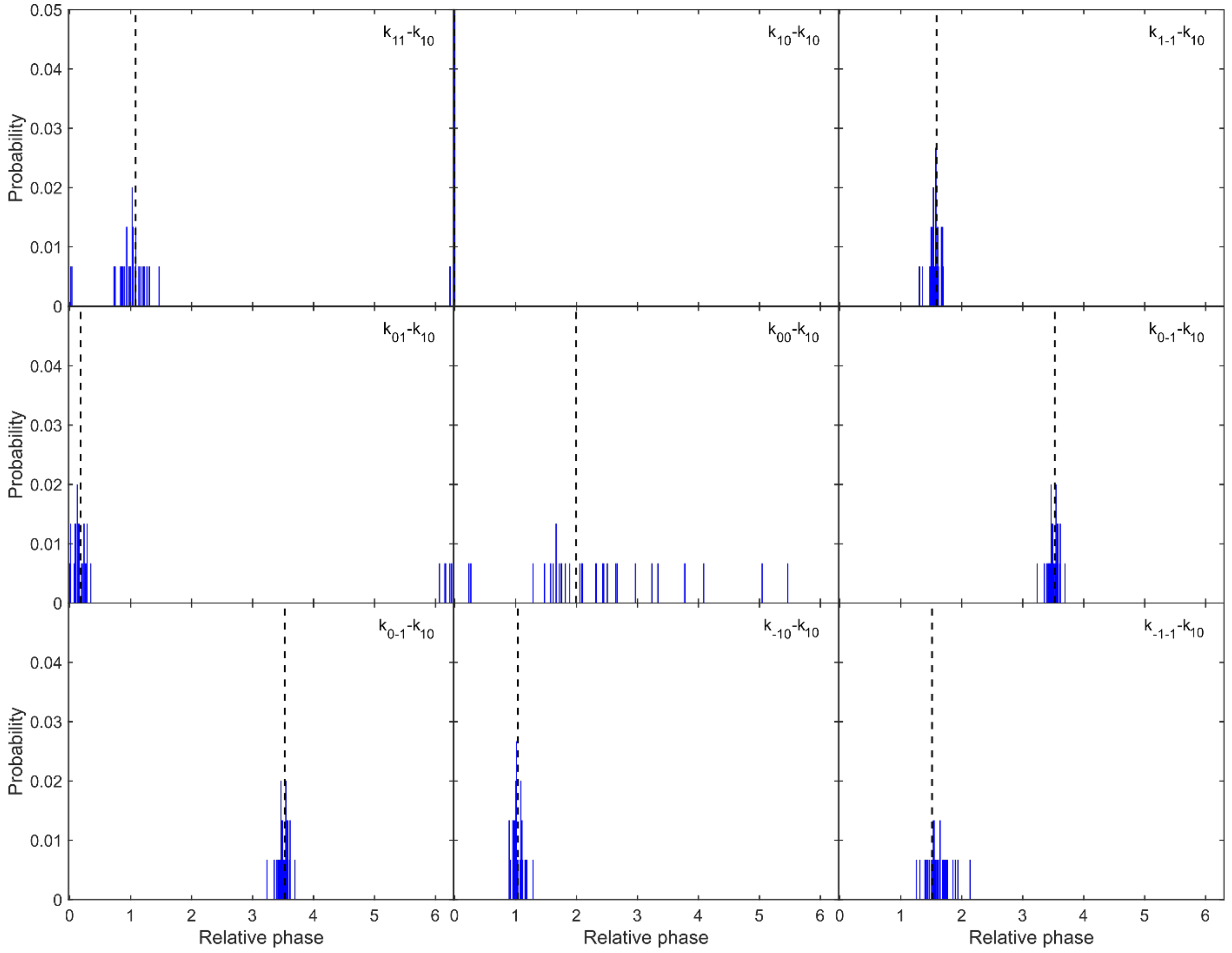

Supplementary Figure 8. Convergence of S-matrix phases. The values of the S-matrix phases (relative to k_{10}) obtained from the best 30 of 150 fits of the simulated data presented in Figs. S6a and S6b, demonstrating the convergence of the 15 parameter fits. The black dashed line in each panel shows the value of the parameter that was used to produce the simulated data. Note that the relatively wide distribution of relative phases obtained for k_{00}-k_{10} coincided with a relatively small amplitude which reduces the sensitivity of the fit to the phase of this element.

Supplementary References

1. Jardine, A. P., Fouquet, P., Ellis, J. & Allison, W. Hexapole magnet system for thermal energy ^{3}He atom manipulation. *Rev. Sci. Instrum.* **72**, 3834–3841 (2001).
2. Utz, M., Levitt, M. H., Cooper, N. & Ulbricht, H. Visualisation of quantum evolution in the Stern–Gerlach and Rabi experiments. *Phys. Chem. Chem. Phys.* **17**, 3867–3872 (2015).
3. Krüger, C., Lisitsin-Baranovsky, E., Ofer, O., Turgeon, P.-A., Vermette, J., Ayotte, P. & Alexandrowicz, G. A magnetically focused molecular beam source for deposition of spin-polarised molecular surface layers. *J. Chem. Phys.* **149**, 164201 (2018).
4. Ramsey, N. F. Theory of molecular hydrogen and deuterium in magnetic fields. *Phys. Rev.* **85**, 60–65 (1952).
5. Godsi, O., Corem, G., Alkoby, Y., Cantin, J. T., Krems, R. V, Somers, M. F., Meyer, J., Kroes, G. J., Maniv, T. & Alexandrowicz, G. A general method for controlling and resolving rotational orientation of molecules in molecule-surface collisions. *Nat. Commun.* **8**, 15357 (2017).
6. Mowrey, R. C. & Kroes, G. J. Application of an efficient asymptotic analysis method to molecule–surface scattering. *J. Chem. Phys.* **103**, 1216–1225 (1995).
7. Cantin, J. T., Alexandrowicz, G. & Krems, R. V. Transfer matrix theory of surface spin echo experiments with molecules. *https://arxiv.org/abs/1906.04846* (2019).
8. Nelder, J. A. & Mead, R. A simplex method for function minimization. *Comput. J.* **7**, 308–313 (1965).
